# Insecticide-treated nets mass distribution campaign: benefits and lessons in Zambia

**DOI:** 10.1186/s12936-018-2314-5

**Published:** 2018-04-24

**Authors:** Freddie Masaninga, Nawa Mukumbuta, Ketty Ndhlovu, Busiku Hamainza, Pauline Wamulume, Emmanuel Chanda, John Banda, Mercy Mwanza-Ingwe, John M. Miller, Birkinesh Ameneshewa, Abraham Mnzava, Elizabeth Kawesha-Chizema

**Affiliations:** 1 World Health Organization, Lusaka, Zambia; 2 School of Medicine, Department of Public Health, University of Zambia, Lusaka, Zambia; 3 Ministry of Health, National Malaria Elimination Centre, Lusaka, Zambia; 4 PATH Malaria Control and Elimination and Partnership in Africa, Lusaka, Zambia; 5 World Health Organization, Africa Regional Office, Lusaka, Zambia; 6 World Health Organization, Africa Regional Office, Brazzaville, Congo; 7 African Leaders Alliance (ALMA), P. O Box 1973, Arusha, Tanzania

**Keywords:** Malaria, Vector control, Mass distribution of LLINs, Impact, Zambia

## Abstract

**Background:**

Zambia was an early adopter of insecticide-treated nets strategy in 2001, and policy for mass distribution with long-lasting insecticidal nets (LLINs) in 2005. Since then, the country has implemented mass distribution supplemented with routine delivery through antenatal care and under five clinics in health facilities. The national targets of universal (100%) coverage and 80% utilization of LLINs have not been attained. Free mass LLIN distribution campaign in Zambia offers important lessons to inform future campaigns in the African region.

**Methods:**

This study reviewed LLIN free mass distribution campaign information derived from Zambia’s national and World Health Organization Global Malaria Programme annual reports and strategic plans published between 2001 and 2016.

**Results:**

In 2014, a nationwide mass distribution campaign in Zambia delivered all the 6.0 million LLINs in 6 out of 10 provinces in 4 months between June and September before the onset of the rainy season. Compared with 235,800 LLINs and 2.9 million LLINs distributed on a rolling basis in 2008 and 2013, respectively, the 2014 mass campaign, which distributed 6 million LLINs represented the largest one-time-nationwide LLIN distribution in Zambia. The province (Luapula) with highest malaria transmission, mostly with rural settings recorded 98–100% sleeping spaces in homes covered with LLINs. The percentage of households owning at least 1 LLIN increased from 50.9% in 2006 to 77.7% in 2015. The 2014 mass campaign involved a coordinated response with substantial investments into macro (central) and micro (district) level planning, capacity building, tracking and logistics management supported by a new non-health sector partnership landscape. Coordination of LLIN distribution and logistics benefited from the mobile phone technology to transmit “real time” data on commodity tracking that facilitated timely delivery to districts.

**Conclusion:**

Free mass distribution of LLINs policy was adopted in 2005 in Zambia. Consistently implemented, has not only contributed to increased coverage of LLINs, but has also produced the added value and lessons of strengthening joint planning, strategic coordination, partnerships with non-health sector institutions and community engagement with traditional leaders at community. Furthermore, the mass distribution, through improving coverage has indirect added (spin-off) value or impact on other arthropod-borne diseases, in addition to malaria.

## Background

The World Health Organization (WHO) recommends full coverage of populations at risk of malaria with effective vector control, which may be achieved through the use of long-lasting insecticidal nets (LLINs) and/or indoor residual spraying (IRS) [1, 2]. Zambia was an early adopter of ITNs for malaria control in the late 1990s with mass distribution policy adopted in 2005. The ITN intervention became one of the most important vector control strategies implemented within an integrated package with IRS in the national malaria strategic plan (NMSP) 2001–2005, with an operational target of three ITNs per household and usage targets of 80% among pregnant women and children under age 5 were adopted [3, 4].

In the first of Zambia’s NMSP 2001–2005, various methods were used to distribute ITNs, targeting different geographic, economic and vulnerable components of the society based on available nets and largely driven by donor funding. By 2005, a “mixed” delivery approach, including mass distribution, was deployed “on a rolling basis” from one district to another based on increasing availability of LLINs, through Global Fund and other partners resources. This was supplemented by continuous distribution through antenatal care (ANCs) clinics and expanded immunization, the promotion of commercial market distribution to ensure wider, long-term sustainability and replacement. An additional effort through the World Bank-supported Community Malaria Booster Response (COMBOR)—a demand-driven strategy to mobilize community activities, including sensitization and net re-treatment campaigns, provided support to improve local awareness and use. The COMBOR replaced the former Community-Based Malaria Prevention and Control Programme (CBMCP) that was based on a push strategy that is, a strategy where ITNs were allocated to communities by the malaria programme by central level planners and implementers based on availability [5].

Prior to mass distribution, only one million ITNs cumulative total was achieved in Zambia in the period 2000 to 2004, with limited impact on the malaria burden. Limited availability of ITNs due to inadequate funding was a major limiting factor to the attainment of increased household ownership of ITNs. By the end of the first NMSP 2001–2005, Zambia adopted the use of LLINs as opposed to conventional mosquito nets that needed re-treatments with insecticides and the national programme had mobilized increased technical assistance, financial and other resources for vector control to scale-up ITNS/IRS [6].

By 2013, annual ITN distributions were about 1.5 million per year and ITN ownership of at least one ITN per household was 72% with utilization among all household members reported at 49%—still substantially below targets for universal (100%) coverage and 80% utilization [7–9]. Noting these gaps and in an effort to accelerate the attainment of universal coverage with LLINs of the national objective of 75% reduction of malaria incidence by 2015, the NMCP in Zambia undertook a one-time comprehensive mass distribution of LLINs in 2014 in line with its NMSP 2011–2016 strategy [10].

The national ITN mass campaign indicated lessons learnt from which other countries in the sub-Saharan Africa embarking on similar interventions could benefit. Here we review the process of LLINs mass distribution during the 2014 campaign and report on the lessons learnt during the 2014 campaign combined with the lessons obtained over the past decade, i.e., since the adoption of the mass ITN campaigns to enable refinement for improved planning, implementation and evaluation of future mass ITN campaigns.

## Methods

Baseline information on free mass distribution, ownership and utilization of LLINs was derived from several sources. The sources formed a basis for analyzing issues related to planning and partner coordination for LLIN mass distribution at all levels of health care—from national down to the sub-national levels (district and community). Key information sources included; National Malaria Strategic Plans [4, 11]; Malaria Indicator Survey reports [7–9], World Malaria Report (WMR) [12, 13] and programme reviews. Progress reports and plans were also reviewed [14–16]. Key milestones were analysed for Zambia’s vector control strategies between 2005 and 2014 (Table 1). The focus was on implementation from the stage of micro-planning, procurement and supply, storage, logistics management, capacity building, community mobilization, mass distribution to end users and partnership involved in LLINs mass distribution (Table 1). To validate the information or data on micro-planning, procurement and supply, storage and logistics, advocacy, partners support and end user distribution various key informants were consulted through the existent vector control technical working members ranging from NGOs to bilateral and multilateral agencies involved in vector control.

**Table 1:** Milestones on mass distribution of long lasting insecticidal mosquito nets in Zambia; 2005–2014

Adoption of mass distribution
Free mass distribution of ITNs commenced in 2000
Policy to use of LLINs rather than conventional nets adopted in 2005
Numbers distributed and ownership
Over six million LLINs distributed in mass campaign within 4 months in 2014 alone compared an annual average of 1.5 million LLINs per year distribution before 2014
Ownership rate of LLINs nationwide: 72% in 2012 and 76% in 2015
LLINs delivery to end user
Door-to-door strategy for delivering LLINs to end user adopted in 2008
Guidelines on the door–door-campaign produced in 2008
Door-to-door strategy revised/refined in 2014 to involve the creation of distribution sites within communities; health workers had to hang the LLIN before leaving house
Record management achieved through use of ITN register maintained by Community Health Workers, ITN agents
Procurement and supply
The World Food Programme (WFP) participation facilitated LLINs logistics management (distribution) from central (national) level to the sub-national level
Direct LLIN distribution from supplier to district piloted in 2004; used in 2014
Direct LLIN distribution from supplier to district—eased storage bottlenecks in 2014
Coordination
The National Malaria Control Programme was responsible for providing overall coordination of the 2014 mass LLIN campaign through use of Technical Working Groups (TWGs)
In 2014, two additional TWGs were constituted to be responsible for the coordination of: (a) procurement, distribution and supply of LLINs (b) monitoring and evaluation of LLINs, including data collection and analyses. The two newly constituted TWGs were an addition to the existent TWG on ITNs and Social Mobilization Behaviour Change and Communication
Partnership landscape
In 2014, to enhance LLINs distribution to end user, World Food Programme (WFP)
Jointed malaria programme partnership, consisting of Churches health association of Zambia (CHAZ), Malaria Control and Evaluation (MACEPA) at PATH funded by Bill and Melinda Gates, JICA, United States President's Malaria Initiative (PMI) through World Vision International and The Global Fund to fight HIV and AIDS, Tuberculosis and Malaria (GFATM) through local funding principal recipients, UNICEF and WHO

## Results

### Micro-planning

The Ministry of Health and partners developed a comprehensive micro-plan for the 2014 mass distribution. This contained details on processes and a roadmap, overall needs (after conducting a rigorous gap analysis) and the procurement plans, roles and responsibilities for national (central), provincial and district level to guide the mass campaign (Table 2). Provincial and district health staff and communities (including area chiefs and traditional leaders) were informed 6 months earlier of the mass campaign plan to conduct mass distribution. This earlier notification fostered ownership and allowed the community leaders, chiefs and volunteers to have adequate planning and orientation in their catchment areas. In addition, several workshops were held with provincial and district planners, and, District Malaria Focal Points to ensure that each province or district clearly understood the scale of logistics to conduct the mass distribution.

**Table 2:** Mass distribution of long-lasting insecticidal nets (LLINs) in Zambia: coordination and key roles by level of implementation, 2014

Level	Coordination body/staff	Role (s)
National	TWGs	Providing overall coordination/oversight
		Mobilizing resources
		Procurement of commodities
		Development of IECs materials
Sub-national		
District	PMO	District planning
		Logistic arrangements
	DMO	District coordination
	MATFS	Supervising Health Centre Committees
Health Centre (HC)	HC Advisory Committees	Mobilizing Communities
		Supporting micro-planning
		Supporting advocacy activities
Community	NHCs	Supporting microplanning
		Mobilizing communities
		Supporting advocacy activities
		Supporting IEC dissemination
		Facilitating household enumeration
		Supporting LLIN distribution

*TWGs* Technical Working Groups, *PMO* Provincial Medical Officer, *DMO* District Medical Officer, *MATFs* Malaria Task Forces, *IEC* Information, communication education, *NHCs* Neighborhood health committees

The National Malaria Control Programme also ensured that community structures such as neighbourhood health committees (NHCs) were linked to the district level during micro-planning to enable accurate estimation of LLINs and the number of days required for the delivery. The mass campaign plan also developed templates that contained information on household sleeping spaces (Table 3), human resource requirements, quantities of mosquito nets, transport, finances for activities and logistics on LLIN distribution required within the district and health centre catchment area as well as a mechanism for coordinating distribution and community engagement at district and community levels. Macro plans were validated by the central level, i.e., National Malaria Control Programme.

**Table 3:** Proportion of sleeping spaces percent coverage with long-lasting insecticidal nets by province and district during mass distribution campaign in 2014, Zambia

Province	District	Target population	Number of sleeping spaces	% coverage*
Central	Chibombo	338,083	177,652	78
	Itezhi-tezhi	132,500	55,300	92
	Kabwe	251,689	156,841	83
	Kapiri mposhi	289,848	276,553	62
	Mkushi	219,480	124,407	83
	Mumbwa	335,692	196,424	77
	Serenje	214,000	111,880	100
Eastern	Chadiza	148,824	81,361	88
	Chipata	643,516	409,826	74
	Katete	288,733	163,177	100
	Lundazi	453,488	258,868	84
	Mambwe	79,795	51,995	92
	Nyimba	108,439	66,443	79
	Petauke	382,743	228,725	92
Muchinga	Chama	114,297	58,444	98
	Chinsali	101,173	59,899	80
	Isoka	96,783	54,009	88
	Mafinga	106,095	57,883	83
	Mpika	311,968	176,225	81
	Nakonde	140,688	88,320	92
	Shiwan’gandu	101,233	52,570	80
Northern	Chilubi	162,100	74,366	68
	Kaputa	153,089	79,904	98
	Kasama	333,406	189,416	87
	Luwingu	174,355	101,389	96
	Mbala	218,545	132,439	98
	Mporokoso	148,223	76,926	89
	Mpulungu	136,315	68,518	99
	Mungwi	171,189	97,863	100
Southern	Choma	256,480	236,554	69
	Gweembe	243,300	26,998	100
	Kalomo	281	210,228	89
	Kazungula	160,764	88,337	85
	Livingstone	157,700	111,321	87
	Mazabuka	315,706	205,706	73
	Monze	273,198	149,813	83
	Namwala	110,396	82,737	83
	Siavonga	110,466	68,110	96
	Sinazongwe	141,693	77,511	88
North-Western	Chavuma	50,373	30,339	76
	Ikelenge	39,855	25,910	79
	Kabompo	121,923	70,403	86
	Kasempa	100,139	59,235	81
	Mufumbwe	90,985	48,878	81
	Mwinilunga	145,070	84,837	81
	Solwezi	272,730	145,998	100
	Zambezi	122,928	65,618	81
Lusaka	Luangwa	31,503	17,715	95
Luapula	Samfya	308,217	182,087	100
	Milenge	59,670	36,725	100
	Mansa	365,801	230,850	99
	Mwense	144,533	99,451	100
	Kawambwa	119,113	75,698	99
	Nchelenge	284,276	170,065	100
	Chiengi	187,674	109,807	100
	Chipili	57,123	34,609	100
	Chembe	62,402	37,725	98
	Lunga	18,036	25,025	100
	Institutions	200,000	136,180	100

No data presented on Western and Copperbelt for sleeping spaces

% coverage* Number of nets distributed divided by number of sleeping spaces

### Procurement and supply

A total of 6,646,500 LLINs were procured for this campaign in 6 out of 10 provinces of Zambia. The other 4 provinces were covered with LLINs during a rolling mass distribution the previous year. Seventy per cent of the total budget of 20.6 million Zambian Kwacha (approximately USD 4.1 million at the exchange rate then) was spent on procurement of LLINs for mass distribution; and, the rest was spent on miscellaneous, including freight, insurance, and programme management, transportation from the central storage (hub) to health facilities and on the distribution from health facilities to end-users. United States dollars (USD) 5.07 per net included; cost of the LLIN plus distribution (to end user) and community mobilization.

**Fig. 1:**
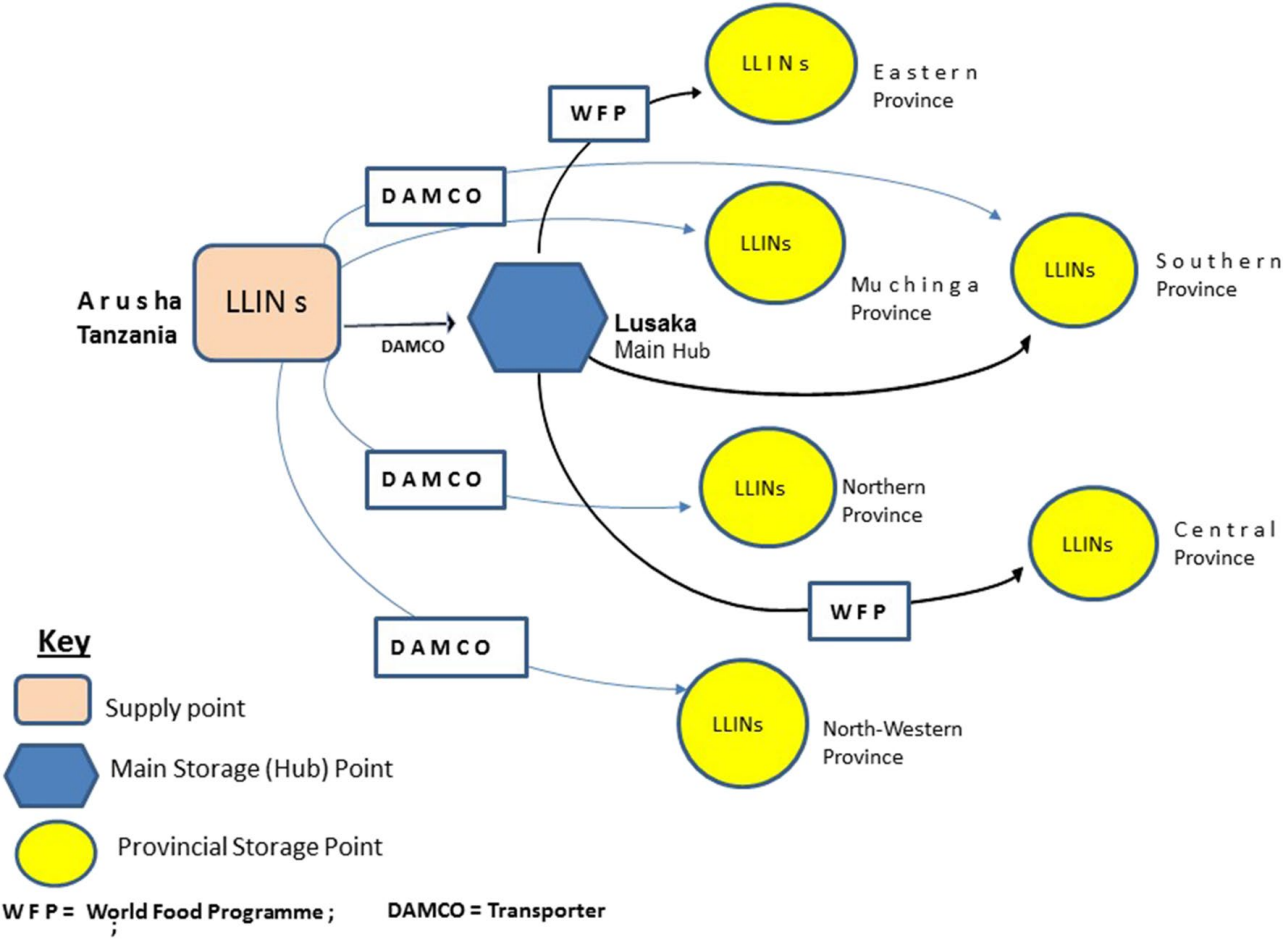
Delivery strategy of insecticidal treated nets (LLINs) during the mass distribution campaign in Zambia 2014

To explore improved efficiency of supply methods, direct distribution from supplier to the district was conducted. However, there were some deliveries which still passed through the national (central) level storage area in Lusaka (Fig. 1). To achieve a direct delivery, the supplier or manufacturer was requested to distribute a quarter of the 6,646,500 LLINs from source of supply directly to districts without passing through central storage in Lusaka, the capital city of Zambia (Fig. 1). This study found that direct delivery eased storage bottlenecks.

Internal quality assurance (QA) was conducted at the factory stage but verifications were done independently facilitated by United Nations Development Programme (UNDP) on behalf of the national health authorities. The internal quality checks were in conformity with WHO recommendations. This internal quality assurance included; checks on physical characteristics-stitching, trimmings, labelling, weights, sizes and cleanliness. Likewise, “external” quality assurance was conducted prior to distribution, 14 samples of LLINs from different batches were sent to a Research Centre in Belgium at Wallon, Gembloux to assess physical and chemical analyses such as stress analysis, insecticide content, isomer ratio, fabric weight, insecticide wash resistance index, netting mesh size, dimensional stability to washing and bursting strength of the net fabric and seams. All the samples conformed to the WHO procurement and use of LLIN for malaria control requirements.

### The mass campaign: storage and logistics

A quarter of the overall 6,646,500 LLINs procured for the mass distribution in 2014 was distributed by the supplier directly from the manufacturer in Arusha, Tanzania to provincial storage areas (hubs) and then to health centres. The remaining 75% of the LLIN consignment was transferred initially to central storage hub in Lusaka (capital city of Zambia) from where the World Food Programme (WFP) transported them to designated network of eight strategic in-country provincial storage areas (hubs) managed warehousing by trained WFP staff. The WFP leveraged 900 trucks from the local truck association. The local truck association was well placed in navigation through tough terrain to remote areas, under tight delivery schedule/timelines to distribute LLINs to 974 health centres for distribution by CHWs to communities. From health facilities the MOH and CHAZ facilitated the door-to-door distributions through the involvement of CHWs. CHWs form a link between local health system and communities.

World Food Programme used new technology—a specialized Relief Item Tracking System (RITA) developed and maintained by WFP logistic staff in Rome, Italy. This mobile-based technology involved assigning a Quick Response Code to each batch of LLINs linked to Geographic Positioning Location obtained at each locality by WFO staff and delivery information. By scanning the Code with a mobile phone, the WFP and partners were able to quickly identify where and when LLIN were dispatched and to transmit daily accurate, well maintain records (data) on LLINs consignments. RITA tracking system facilitated the step-by-step tracking of LLIN movement on each route for each truck from central warehouse to the health facility. Thus, WFP ensured availability timely, of LLINs at the destination point, in good condition, in right quantities with minimum loses while in transit (only about 0.08%).

### Preparations for distribution at community level

Household data for each catchment area, including sleeping spaces registered collected from community registers, were used to develop a detailed district micro plan that indicated the needed human resources, quantities of mosquito nets, transport, finances and other logistics required for each health centre catchment area. To avoid discrepancies in the LLINs requirements for each household, rigorous verification of the LLIN data was done for each district in collaboration with health centre before LLINs were transported from the health centres to various point of use by CHWs. In terms of field validation, data used was at the household level; it included data elements such as name, village, number of persons in each household and the LLINs required. The districts in these provinces were engaged to show examples on any erroneous entries that were subsequently corrected in the registers and the database. An additional 5% random re-sampling was conducted to validate this data. The register were then returned to the health facilities and used as a basis for distribution of the LLINs to the households, indicating how many LLINs were given to each household. These were then re-entered into the excel database and a comparison of need that was actually received was conducted to estimate the LLIN gap by household. These efforts were facilitated by a multi-disciplinary Monitoring and Evaluation Technical Working Group that supported analyses and advice.

### Coordinating and tracking distribution

To ensure effective coordination of the mass distribution efforts, the National Malaria Control Programme (NMCP) plan involved constituting coordinating committees, specifically for the mass ITN campaigns with defined terms of reference (TORs), mainly focusing on ensuring smooth operations of the procurement, commodity tracking, distribution, monitoring and evaluation, community mobilization/sensitizations and holding of a series of meetings before, during and after the mass distribution campaign. The new committees (Table 2), worked with the already existent structures in the NMCP such as ITNs, M&E, and IECs Technical Working Groups (TWGs) at national, sub-national or provincial level to community level to implement the planned activities.

At the sub-national level, coordination was mediated through the Provincial Medical Officers (PMOs) and the District Medical Officer (DMOs) working with district-level Malaria Task Forces (MATF). MATF consisted of heads of Government departments, Local Municipal Council authorities, parastatal bodies, business community and non-governmental (NGOs), including Churches Health Association of Zambia (CHAZ). The PMOs supported development and implementation of the micro-plans, logistics and administrative arrangements required for effective delivery of LLINs to communities, while the DMOs worked with MATFs to coordinate implementation at the district level. At the health facility level, Health Centre Advisory committees linked the district MATFs with community structures (neighbourhood health committees, area chiefs and religious leaders) to mobilize full community participation or action that included enumeration of households, door-to-door distribution of LLIN, and delivery of key messages to promote effective use and discourage misuse of LLINs. At the community, area chiefs, traditional leader’s support was particularly valuable.

The use of mobile phone technology enabled monitoring of progress on LLIN distribution in all areas including, hard-to-reach areas at subnational level which had previously not received real time monitoring. Community volunteers at each health center collected data on a number of households, sleeping spaces and the number of LLINs required at each household. The data was aggregated and sent through health facilities to district office where it was further validated and submitted to the national (central level) for use in coordinating the mass distribution and production of the final report.

### Capacities for LLIN mass distribution

The National Malaria Control Programme staff trained in ITNs for malaria vector control/elimination and logistics management supported by partners served as master Trainers of Trainers (ToTs) of district staff who in turn cascaded training to health facility and community levels. Sixty-four Provincial and 1161 district staff were trained including, planners, environmental health technologist, nurses and clinical officers in-charge of operations at health facilities. At the district the training initially started with health centres-in-charge who then cascaded it to CHWs; the cascades helped to train, coordinate and supervise community volunteers from neighbourhood health committees. The training also provided skills on registration of households, data collection tools, and data verification using national malaria training manuals adapted from WHO guidelines built on lessons obtained from other national malaria programmes in the African sub-region.

Immediately after training community members were provided with information, education and communication materials which they used to deliver key malaria messages. They were also provided with registration forms for the them to use in registering sleeping spaces in the household in their respective catchment areas.

### Synergies among partners in the mass campaign

Each partner brought unique comparative advantages to the mass campaign. The United Nations Children Fund (UNICEF) facilitated LLINs procurements given their expertise in long-term agreements with WHO-certified pharmaceutical product suppliers. UNICEF also financed the development of malaria messages and radio programmes during and after distribution. The UNDP strength in project management, supported MOH’s coordination/management of the whole process. WHO’s strengthen in development of strategic documents and guidelines coordinated the development of comprehensive technical and financial gap analysis for the LLINs in collaboration with the US President’s Malaria Initiative, Malaria Control and Elimination Partnership in Africa and other partners. The gap analysis was built on various assumptions that took into account several factors including, target population for the LLINs, source of funds (assured or not), unit cost for the delivery of one LLIN to the household. The gap analysis also took into account the average user-life of an LLIN, costs for community mobilization, IECs production and advocacy. To enhance quality delivery, the gap analysis was validated through a peer review team of experts and consultants from the regional and global malaria partnership. These were skilled in project planning, procurement, supply and distribution management. The use of Gap Analysis Excel spreadsheet (tool) has over the past 5 years evolved. The WHO Global Malaria Programme has provided technical support which has strengthened capacities for vector prevention commodity, case management and programme gap analyses in the malaria programme in Zambia.

The engagement of WFP, an agency with strong logistics and transport knowledge obtained over many years when dealing with relief food to remote hard to reach areas, freed the malaria programme from complexities of commodity (LLIN) distribution and transportation responsibilities and challenges to concentrates on providing overall oversight. The WFP delivered LLINs from point of manufacturer in Tanzania to the health facility level. Government ministries including; the Ministry of Education, Ministry of Defense and the Ministry in charge of Community Development and local municipal authorities supplemented transport needs.

Local District administrators—such as the District Commissioners (DCs) supported district coordination meetings. The Churches Health Association, a Faith-Based Organizations (FBO) and World Vision International with vast experience in working with CHWs in rural, hard to reach communities worked with Community NHC structures to achieve “the last mile” distribution of LLLINs through the door-to-door LLINs delivery to beneficiaries. Community NHC structures were particularly critical in the final distribution of LLINs from Health facilities to end users; they supported correct LLIN use.

### The mass LLINs distribution campaign

**Fig. 2:**
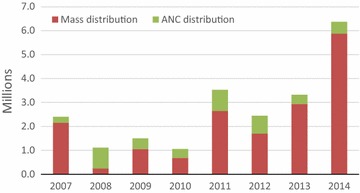
LLINs Mass and ANCs distributions absolute numbers

A total of 6,646,500 LLINs were procured for the mass distribution (Fig. 2). Of these 6.0 million were distributed through mass distribution to the beneficiaries, while the 646,500 (balance from the procurement) was kept to be used for routine distribution. The Global Fund against HIV/AIDS, tuberculosis and malaria procured 4,725,357 LLINs; the United States Presidents Malaria Initiative procured 1,627,357 LLINs; the Ministry of Health and NGOs contributed 16,673 and 277,113 respectively. With micro-plans developed and shared at all levels, implementation of the mass distribution campaign was smooth. In Zambia, the allocation of an LLIN to a household to achieve universal coverage with LLINs was based on delivering one LLIN per sleeping space in a given household; one net for every two people allocation was used in the previous year.

### Door-to-door distribution vs fixed point distribution of LLINs

**Fig. 3:**
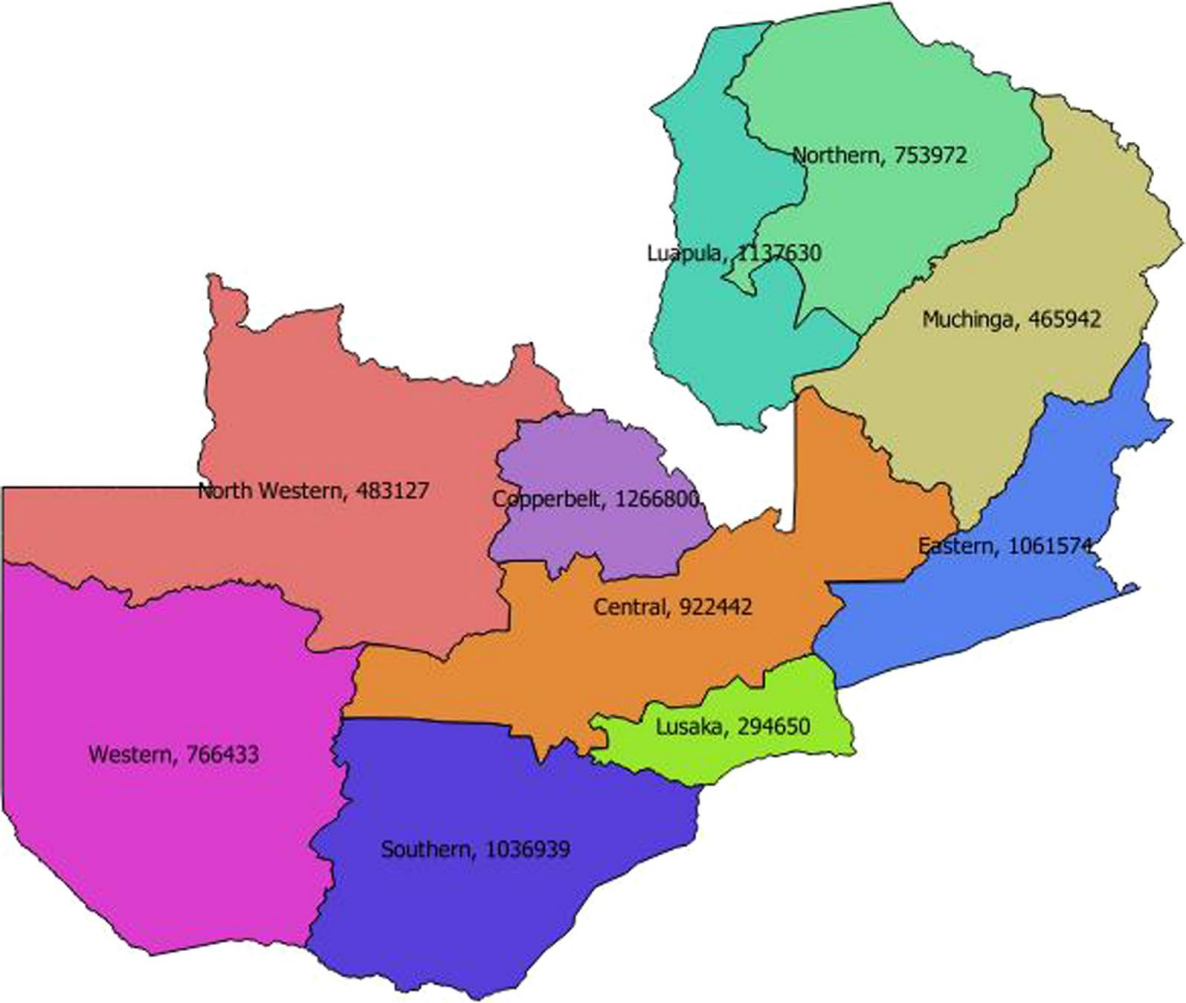
Map LLIN distribution by province

The majority (90%) of the LLINs were delivered to beneficiary households through the door-to-door method according to an approved distribution plan. By this method (i.e., door-to-door) a CHW directly delivered LLINs to recipients at their homes, hung the nets over each sleeping place for them and provided the beneficiaries with key messages and package of IEC for behaviour change and communication in the different provinces (Fig. 3) before leaving the households. Village-community registers were used for the distribution of LLIN to households. This provided a systematic way of distribution, thereby avoiding giving LLINs to persons who could have already received the nets. The distribution of LLINs to the remaining 10% of households was through—the “fixed point” distribution, another method where LLINs were delivered to the beneficiary at a selected, fixed point—usually a school or church premise in situations where heads of household (house owners) did not accept/allow entry of net distributors into their bedrooms or sleeping place areas due to cultural reasons.

Information, Education, Communication materials and health education were also provided at the “fixed point” distribution sites. The actual numbers of LLINs for distribution during the each of the two methods was guided by the district micro-plan, supported by national guidelines developed to support door-to-door and fixed point.

### Continuous LLINs distribution complementarity

**Fig. 4:**
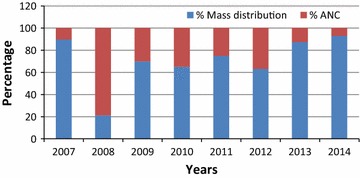
Percentage of LLINs distributed by mass and ANC distributions

In Zambia, implementation of the mass LLINs distribution has been complemented by routine distribution through antenatal clinics (ANCs) to sustain achieved ownership and use by mass distributions (Figs. 2, 3 and 4). In 2007, a total of 248,655 LLINs were delivered through antenatal care clinics. The number increased to 885,500 in 2011 (about 25% compared mass distribution) but ANC distribution was reduced in 2014 to only 490,000 LLINs because of increased volume distributed through the mass campaign (Fig. 2). During the first National Malaria Strategic Plan in 2001–2005 school distribution was employed as a complimentary approach to service the school-going population but was discontinued in the NMSP 2006–2010 due to competing budget needs for integrated vector control in the country. An attempt to revive this is currently underway.

### Information education communication (IECs) and community mobilization

Community mobilization before, during and after the mass LLIN distribution campaign was mediated through the use of various channels that included interpersonal communication, mass and the print media. To assure quality, harmonization and coordination, key messages were developed by the national IEC Technical Working Group team of experts derived from several partner agencies and NGOs. This team developed a comprehensive IEC package containing, radio guides, scripts for radio stations, ITNs leaflets. The materials, which included brochures and fliers, were used to provide information on the planned mass campaign, solicit community participation and inform on the need for sustained use of LLINs.

It was observed that radio broadcasts on key ITN messages facilitated a highly valuable interaction between health workers staff and communities. The radio messages were produced both in English and local languages which was appreciated by communities. In the past 5 years several radio stations have been established in all the provinces at the district level in Zambia. Broadcast on local radio stations using “phone-in radio programmes” helped ITN district programme managers to respond to questions raised by communities on the distribution process on a real-time-basis and to remove any negative rumours. Broadcasts reinforced critical information on ITN use and care to strengthen general community awareness on malaria.

The NMCP delivered the health promotion messages and materials to Provincial Offices for final dissemination to communities. Training related to the IEC for behavioral change was integrated into the main LLINs cascade training to equip Community Health Workers on interpersonal communication skills, focusing on effective use of LLINs by householders. CHWs with support from area traditional leaders (chiefs) delivered key LLIN messages strategically identified periods or phases of the mass campaign, namely; before and during the mass campaign to inform communities on the value of participating in the mass campaign and the need for them to spear-head the education campaign, given their comparative advantage of living within the community and personal relationship with the beneficiaries. The CHWs also played the role of ensuring that the nets that were hung, were being utilized; they also answered any questions that beneficiaries raised.

## Discussion

A free mass distribution campaign conducted in 2014 in Zambia delivered about 6 million LLINs to communities at risk of malaria infection and offered important lessons. The benefit of a timely and comprehensive micro-planning at national and sub-national (provincial and districts) levels that characterized mass campaigns was particularly evident. It enabled accurate and efficient estimation of LLINs needs required for smooth implementation of the mass campaign. Early involvement and cooperation by the different levels at micro-planning stage proved useful.

An important challenge acknowledged by the Roll Back Malaria Tool Kit on LLIN mass campaign by the Alliance for malaria prevention [24] is that few countries have experience of filling the LLIN gaps required for reaching Universal Coverage with LLINs. In the Zambian mass campaign a significant amount of time was invested in developing the programmatic LLIN gap analysis in collaboration with national, regional expertise and consultants.

The programmatic and financial gap analysis on LLINs needs coordinated by the national level but implemented by respective districts served as an important tool for quantification of the LLINs needs or gaps and provided a transparent, accountable tool for planning; it also encouraged ownership in planning by the participating districts and various partners providing funding or technical assistance. The processes on mass distribution from central level micro planning to the community, coordinated through a monitoring and evaluation committee benefited from the use of mobile phone technology to track LLIN distribution for a timely delivery of commodities to districts.

Another important lesson from the mass distribution was on procurement and supply of LLINs to the end user, related to involvement of the World Food Programme. The WFP expertise in logistic management enabled reliable, accountable, efficient transportation; warehousing, commodity tracking and logistics management that enabled efficient transportation of LLINs close to beneficiaries on-time in remote hard-to-reach areas. These capacities coupled with the use of electronic mobile phone technology system to manage records on LLINs consignments enabled effective coordination of the mass campaign from the national to the sub-national levels. This review demonstrates the opportunities offered through mass distribution in fostering viable collaboration between health and non-health institutions/agencies. The use of direct-manufacturer-to-districts distribution (without passing through central warehouse) eased logistics bottlenecks that were encountered during previous distribution through central storage/warehousing prior to their dispatching to districts.

Partnership for the 2014 mass campaign was broad and strategic. It included; research institutions and academia, United Nations agencies, US-President’s Malaria Initiative, PATH-Malaria Control and Elimination Partnership in Africa. The strategic use of NGOs such as Churches Health Association of Zambia (CHAZ) and other non-governmental organizations (World Vision), traditional leaders and neighbourhood health structures permitted timely distribution of the LLINs to remote rural, hard-to-reach areas having difficult terrain in rural districts. CHAZ penetrates deep into rural remote areas hence enabling successful delivery of LLIN to the householder. Government ministries such as the Ministry of Education, Ministry of Defense and Community Development played a vital role in supplementing transport and other logistics requirements additional to that provided through WFP.

The door-to-door delivery of LLINs to households allowed higher coverage of hard-to-reach areas. It also provided an opportunity for demonstrating net-hanging and face-to-face health education on LLIN use and ways of reducing net wear and tear. Fixed point distribution was used in situations where communities where reluctant to allow CHWs to enter their sleeping places (houses) due to cultural beliefs.

Investment into the development of key message to disseminate information and their appropriate delivery through inter-personal communication, mass and print media coupled with hands-on instruction to householders on net-hanging, maintenance helped to increase community awareness and uptake of the malaria interventions. In 2015, the proportion of women aged 15–59 who reported mosquito net use as a preventive method against malaria was 91% up from 80% reported in 2013–14, attesting to the value of investment into household awareness creation [10]. The database showed that areas/districts experiencing the highest malaria transmission in Luapula province had 98–100% of the sleeping spaces in the household covered with LLINs. A nationally representative malaria survey conducted after the mass campaigns showed significant progress in LLIN ownership made in Zambia, with 77% households owning at least one ITN and 80% of the households owning at least one mosquito net (any net) and approximately 59% (the proportion of ITN among the 10 provinces of Zambia ranges from 42 to 79.9%) [10]. In addition to the increasing ITN ownership the country has recorded equitable access to the LLIN between rural and urban areas. Thus access to LLINs in rural areas increased to almost 80% in 2015 from 63 in 2008. There were also increases in ITN-to-sleeping-space ratios for rural areas (32.5 in 2008 to 63.9% in 2015) and urban areas (28.7% in 2008 vs 56.5%). Moreover, improved equity across gender and wealth, economic groups, with ownership in the lowest quintile being at 77% and ownership in the highest quintile reported at 73% in 2015 [8–10, 17].

The value which the mass campaigns for LLINs offers (in terms of increasing net ownership) corroborates findings from multi-country studies in different African countries, including Nigeria, Ghana and Uganda, where mass campaigns increased ownership of at least one ITN irrespective of a strategy (i.e., whether stand-alone or combined with ANCs) [18]. This multi-country study further showed that delivery, distribution or allocation strategy was not associated with receipt of at least one ITN, and that mass campaign for universal coverage allocation, especially based on the sleeping space allocation were more effective in increasing the proportion of households with adequate ITN [18]. The value of mass ITNs has been recognized previously in other studies in the African region [19]. Pioneer studies conducted in Ghana and Zambia have also shown that integrating ITN distribution into measles vaccination campaign increased levels of ownership, equity at a low cost to the provider and consumer [20]. In the study the ratio of ITN ownership in poorest household compared with least poor rose from 0.32 to 0.88, with the cost per child reducing from 0.89 to 0.57 [20].

Mass campaigns are a vital intervention not only for malaria prevention but for other arthropod-borne diseases, such as filariasis and viral infections whose distribution overlaps with that of malaria as documented in the African sub-region including Zambia [21, 22]. A recent study in Zambia demonstrated significant decline in lymphatic filariasis associated with nationwide scale-up of insecticide treated nets, attesting to the added (spin-off) value of the use of LLINs against other arthropod-borne diseases in the African region [22].

As national disease programmes continue to benefit from LLIN use, they should recognize the challenges and potential risks associated with the use of this intervention. One such challenge is the development of vector resistance to insecticides (pyrethroids), which has been documented in various countries including Zambia, with the potential to reverse the gains made through vector control [23]. Unfortunately, options for insecticide in use for LLINs are limited. The developments of new formulations in combination with non-pyrethroid insecticides to mitigate the challenges in vector control are being explored as measures to address these challenges.

Regular reviews on mass campaigns are vital as they avail critical information that could be used to feed into important existent Tool Kits on LLIN mass campaigns for malaria prevention when a time comes for updating these tool kits [24].

This study highlights the value of implementing a preventive intervention consistently for a long time (decade) [25] that includes: harnessing strategic coordination; partner comparative advantages in LLINs logistics management; micro-planning and community mobilization based on existent community structures; using community these structures, leaders; area chiefs and volunteers to increase nationwide household ownership. Clear policy on LLINs, strategies and targets for vector control at all levels anchored on the malaria strategic plans since 2000 have also been an important enabler in the scale up and significant uptake of LLINs in the country.

## Conclusions

Free mass distribution of LLINs policy was adopted in 2005 in Zambia. Consistently implemented, has contributed to increased coverage of LLINs, but has produced the added value and lessons of strengthening joint planning, strategic coordination, partnerships with non-health sector institutions and community engagement with traditional leaders at community level. Furthermore, the mass distribution, through improving coverage has indirect added (spin-off) value or impact on other arthropod-borne diseases, in addition to malaria.

## Abbreviations

CHW: Community Health Worker
The GF: The Global Fund to fight HIV/AIDs, Malaria and Tuberculosis
NHC: neighbourhood health committees
NMCP: National Malaria Control Programme
LLINs: long-lasting insecticide-treated nets
ITNs: insecticide-treated nets
IEC: Information, Education, Communication
PMI: President’s Malaria Initiative
UNICEF: United Nations Children’s Fund
JICA: Japan International Cooperation Agency
km: kilometre
WFP: World Food Programme
